# Criteria for differentiating left bundle branch pacing and left ventricular septal pacing: A systematic review

**DOI:** 10.3389/fcvm.2022.1006966

**Published:** 2022-09-30

**Authors:** Kailun Zhu, Linlin Li, Jianghai Liu, Dong Chang, Qiang Li

**Affiliations:** ^1^Department of Cardiology, Xiamen Cardiovascular Hospital of Xiamen University, School of Medicine, Xiamen University, Xiamen, China; ^2^School of Medicine, Xiamen University, Xiamen, China

**Keywords:** left bundle branch pacing, left ventricular septal pacing, QRS complex, electrocardiogram, electrophysiology

## Abstract

**Background:**

As a novel physiological pacing technique, left bundle branch pacing (LBBP) can preserve the left ventricular (LV) electrical and mechanical synchronization by directly capturing left bundle branch (LBB). Approximately 60–90% of LBBP were confirmed to have captured LBB during implantation, implying that up to one-third of LBBP is actually left ventricular septal pacing (LVSP). LBB capture is critical for distinguishing LBBP from LVSP.

**Methods and results:**

A total of 15 articles were included in the analysis by searching PubMed, EMBASE, Web of Science, and the Cochrane Library database till August 2022. Comparisons of paced QRS duration between LVSP and LBBP have not been uniformly concluded, but the stimulus artifact to LV activation time in lead V5 or V6 (Stim-LVAT) was shorter in LBBP than LVSP in all studies. Stim-LVAT was used to determine LBB capture with a sensitivity of 76–95.2% and specificity of 78.8–100%, which varied across patient populations.

**Conclusion:**

The output-dependent QRS transition from non-selective LBBP to selective LBBP or LVSP is direct evidence of LBB capture. LBB potential combined with short Stim-LVAT can predict LBB capture better. Personalized criteria rather than a fixed value of Stim-LVAT are necessary to confirm LBB capture in different populations, especially in patients with LBB block or heart failure.

## Introduction

Left bundle branch pacing (LBBP) is a novel physiological pacing technique, in which the active fixation pacing lead delivered by the pre-shaped sheath advanced *via* a trans-ventricular septal approach to directly capture the proximal left bundle branch (LBB) or its branches underneath the left ventricular (LV) septal endocardium to preserve the normal sequence of LV electrical activation and mechanical contraction (1). LBBP can be divided into selective LBBP (SLBBP) and non-selective LBBP (NSLBBP) depending on whether or not the septal myocardium around the LBB is captured (2).

The implantation process of LBBP and LVSP is similar in that both of them are advanced from right ventricular (RV) septum *via* a trans-ventricular septal approach to LV septum, and both of them can produce relatively narrow paced QRS duration and continuously dynamic changes of paced QRS morphology from left bundle branch block (LBBB) to right bundle branch block (RBBB) pattern. LVSP, on the other hand, is fairly straightforward because there is no need to confirm the LBB capture by recording of LBB or His bundle potential, accurate initial pacing localization on fluoroscopy, or extra pacing maneuvers (3, 4). Approximately 60–90% of LBBP were confirmed to have captured LBB during implantation, implying that up to one-third of captures from the left septum are actually LVSP (5, 6). Currently, some researches have provided reliable strategy for distinguishing LBBP from LVSP, with sensitivity ranging from 70 to 100% and specificity reaching 100% (3, 6, 7). In this review, we will focus on the electrical differences between LBBP and LVSP, as well as describe the electrophysiological and electrocardiographic criteria for differentiating LBBP and LVSP.

## Search strategy and outcomes

Electronic databases, including PubMed, EMBASE, Web of Science, and the Cochrane Library database were comprehensively searched (until August 2022) to identify primary references using the terms of (1) “left bundle branch pacing” OR “left bundle branch area pacing” and (2) “left ventricular septal pacing.” We excluded animal studies, abstracts, reviews, editorial and individual case reports. References from the relevant articles were reviewed and related articles were identified. A total of 15 articles were selected for detailed review (3–17) (Table 1).

**Table 1:** Study characteristics of included studies.

**Study**	**Patient number**	**Indication**	**LBB captured n (%)**	**Paced QRS duration (ms)**			**Stim-LVAT (ms)**		
				**LBBP**	**LVSP**	***P***	**LBBP**	**LVSP**	***P***
Qian et al. (7)	68	Bradycardia	47 (69%)	113.4 ± 9.8	120.7 ± 10.7	0.005	None	None	None
Zhang et al. (8)	106	Bradycardia	78 (74%)	115.0 ± 9.4	126.6 ± 12.5	<0.01	70.8 ± 5.7	83.3 ± 7.8	<0.01
Jastrzebski et al. (6)	468	Bradycardia and/or HF	124 (26%)	154.5 ± 21.2 (NSLBBP)	159.3 ± 20.2	None	74.7 ± 12.0 (NSLBBP)	None	None
				175.8 ± 26.5 (SLBBP)			74.4 ± 13.0 (SLBBP)		
Heckman et al. (5)	50	Bradycardia, AVN ablate	31 (62%)	123 ± 22	None	None	73 ± 15	81 ± 13	0.138
Wu et al. (3)	None	Bradycardia and/or HF with LBBB	30 (21 of non-LBBB; 9 of LBBB)	134.3 ± 14.9 (non-LBBB)	141.7 ± 16.6	0.003	70.7 ± 7.7	90.8 ± 15.2	<0.001
				138.4 ± 15.4 (LBBB)	144.7 ± 14.0	0.027	81.7 ± 8.4	97.4 ± 13.1	<0.001
Curila et al. (12)	68	Bradycardia	None	104 (100, 108)	103 (100, 107)	>0.05	70 (66, 73)	86 (84,89)	<0.01
Curila et al. (13)	96	Bradycardia	57 (59%)	LBBP <LVSP (non-quantitative)		<0.001	68 (65, 71) (NSLBBP)	86 (83, 89)	<0.001
							70 (67, 73) (SLBBP)		
Vijayaraman et al. (14)	32	LBBB	25 (78%)	141 ± 15	None	None	75.2 ± 8.8 (NSLBBP)	90.4 ± 9.1	<0.001
							76.9 ± 8.3 (SLBBP)		
Jastrzebski et al. (9)	468	Bradycardia and/or HF	124 (26%)	154.5 ± 21.2 (NSLBBP)	159.3 ± 20.2	None	78.4 ± 10.8 (NSLBBP)	98.4 ± 13.9	None
				144.5 ± 24.4 (SLBBP)					
Shimeno et al. (10)	51	Bradycardia without LBBB	21 (41%)	137 ± 9 (NSLBBP)	135 ± 7	>0.05	60 ± 4 (NSLBBP)	76 ± 7	<0.01
				154 ± 11 (SLBBP)			60 ± 4 (SLBBP)		
Chen et al. (4)	43	Bradycardia without LBBB	27 (63%)	135.6 ± 10.9	141.6 ± 13.6	0.118	65.8 ± 8.1	81.6 ± 7.3	<0.001
Peng et al. (15)	59	Bradycardia	46 (78%)	105.3 ± 15.6	109.2 ± 9.6	0.287	72.0 ± 10.0	86.4 ± 12.3	0.001
Zhou et al. (16)	46	Bradycardia and/ or HF	23 (50%)	104.26 ± 19.00	118.09 ± 23.20	0.032	48.70 ± 13.67	58.70 ± 13.67	0.032
Shimeno et al. (11)	126	Bradycardia	52 (41%)	135 ± 16 (NSLBBP)	141 ± 16	None	62 ± 9	72 ± 10	<0.001
				150 ± 22 (SLBBP)					
Qian et al. (17)	118	Bradycardia and/or HF ± LBBB	90 (76%)	115.9 ± 20.3 (Bradycardia)	135.3 ± 21.0	0.004	67.8 ± 7.8	80.9 ± 10.4	<0.001
				136.0 ± 14.4 (HF ± LBBB)	152.2 ± 15.9	0.005	77.1 ± 8.8	94.6 ± 10.4	<0.001

LBB, left bundle branch; Stim-LVAT, stimulus artifact to left ventricular activation time in lead V5 or V6; LBBP, left bundle branch pacing; NSLBBP, non-selective LBBP; SLBBP, selective LBBP; LVSP, left ventricular septal pacing; AVN, atrioventricular node; HF, heart failure; LBBB, left bundle branch pacing.

## Physiology and practicality of LVSP

Back in 1970, Durrer et al. measured the total excitatory process of seven isolated normal human hearts by as many as 870 intramural terminals (18). Within 5 ms of the occurrence of the LV action potential, the three LV endocardial areas were first activated synchronously that were high on the anterior para-septal wall just below the attachment of the mitral valve, central on the LV septal endocardium, and posterior para-septal about one-third of the distance from apex to base. The excitatory propagated rapidly across these three areas during the following 5–10 ms, and fusing by 15–20 ms (18). Pacing in these first activated areas of the LV septal endocardium can thus be expected to obtain the intrinsic physiological excitation sequence. Subsequently, Little et al. demonstrated in 1982, using echocardiography on nine open-chest dogs, that pacing from the left side of the interventricular septum exhibited the identical sequence of interventricular septal excitation and motion as the intrinsic sinus rhythm (19). Peschar et al. investigated LV systolic and diastolic function using pressure-volume relations with normal sinus rhythm, LVSP, conventional RV pacing, various epicardial sites pacing, and combinations of pacing schemes, and found that LVSP could best maintain normal LV pump function, which possibly due to LVSP producing physiological electrical propagation (19).

In 2016, Mafi-Rad et al. studied LVSP in sick sinus syndrome (SSS) patients with normal cardiac structure and found that LVSP had an immediate effect on LV hemodynamics comparable to atrial pacing and superior to RV apex pacing (RVAP) and RV septal pacing (RVSP) (20). Furthermore, LVSP has a shorter pacing QRS duration than RVAP and RVSP (144 ± 20 vs. 172 ± 33 vs. 165 ± 17 ms, *P* = 0.02 and 0.004, respectively) (20). At 6 months of follow-up, pacing parameters of LVSP remained stable with no lead-related complications. Recently, it was demonstrated that LVSP provides outstanding electrophysiological and hemodynamic performance in patients undergoing cardiac resynchronization therapy (CRT) indications, at least as well as conventional biventricular pacing (BVP) and potentially His bundle pacing (HBP) (21).

## Physiology and practicality of LBBP

LBB, originating in branching portion of the His bundle located underneath the junction of the non-coronary cusp and the right coronary cusp of the aortic valve, distributed in a broad ribbon-like structure in the LV septal sub-endocardium (22, 23). LBB has two main fascicles, the slender left anterior fascicle that heads the anterior papillary muscle and the thick left posterior fascicle (LPF) that heads the posterior papillary muscle of the mitral valve. Furthermore, virtually all of the LV septal fibers, which originate from LPF, were interlaced into a network that radiates to the inferior third of endocardium on left side of the interventricular septum (23). Because of the abundant interfascicular network connections of LV septal fibers, it is possible that when one of the fascicles is blocked, the QRS duration is not significantly prolonged. The ribbon-like structure and interfascicular network connections of LBB make LBBP implantation easier than HBP.

LBBP has electrophysiological advantages over HBP in addition to anatomical advantages. According to the longitudinal dissociation theory, LBB and RBB have been predominantly separated by the insulated fiber sheath inside His bundle (22, 24, 25). The majority of bundle branch blocks may be in the main bundle branch within His bundle. Narula et al. normalized the bundle branch block with distal HBP, shortening the intrinsic prolonged HV interval by 20–35 ms (24). Upadhyay et al. used LV septal mapping in LBBB patients, and concluded that the site of block of complete LBBB was at the level of left-sided His bundle in 72% and in the LBB trunk in the others (26). This provides an electrophysiological basis for LBBP, allowing it to capture the LBB immediately beyond the conduction block with a low output (27), overcoming the limitations of HBP with high pacing output and even loss of capture (28, 29).

In 2017, Huang et al. performed LBBP on a patient with dilated cardiomyopathy and heart failure whose LBBB was not corrected by HBP. The typical LBBB with QRS duration of 180 ms was corrected by LBBP beyond the conduction block with a low pacing output, and then the accompanying RBBB morphology was eliminated by adjusting the atrioventricular delay (27). A number of studies have demonstrated the long-term safety, stability and superiority of LBBP as a physiologic pacing strategy for high-degree atrioventricular block, SSS, and atrioventricular node ablation, etc. (30–37). LBBP has also proved to be a promising method for delivering CRT for typical LBBB patients with low LV ejection fraction, improving heart failure symptoms and LV function greater than conventional BVP (38–40).

## LBBP vs. LVSP

In theory, LBBP rapidly conducts electrical excitation through the intrinsic His-Purkinje system, accelerating the process of LV lateral wall depolarization while comparatively delaying the RV excitation, resulting in electrical dyssynchrony between the left and right ventricles (12, 13). LVSP, unlike LBBP, only captures the LV septal myocardium, and the electrical excitation in the interventricular septum is transversely conducted at the same time, so that the LV delayed excitation partially overlaps with the RV delayed excitation, resulting in a more balanced but non-physiological synchronization of the interventricular electrical excitation (2, 41).

According to the current study, the short-term effects of LBBP and LVSP on cardiac function seem to be less substantial in practice (4–6, 9, 10, 12, 13, 16). There was no consistent outcome in terms of paced QRS duration, although practically all investigations demonstrated that the stimulus artifact to LV activation time (LVAT) in lead V5 or V6 (Stim-LVAT) of LBBP was shorter than that of LVSP (Table 1). Shimeno et al. showed that the paced QRS duration of NSLBBP and LVSP was similar (135 ± 7 vs. 137 ± 9 ms, *P* > 0.05), while capturing LBB reduced the Stim-LVAT by about 10 ms or more (76 ± 7 vs. 60 ± 4 ms, *P* < 0.01) (10). Curila et al. showed no significant difference in the paced QRS duration between LBBP and LVSP [104 (100, 108) vs. 103 (100, 107) ms, *P* > 0.05], similar to the results reported by Peng et al. (105.3 ± 15.6 vs. 109.2 ± 9.6, *P* = 0.287) (15). However, in another study by Curila et al., the paced QRS duration of LBBP was shorter than that of LVSP in close proximity to LBB (13). A retrospective study by Zhou et al. found that LBBP and LVSP had stable pacing parameters and no significant difference in LV function improvement. However, LVSP has the advantage of shorter implantation time than LBBP (38.13 ± 11.52 vs. 53.52 ± 14.39 min, *P* < 0.001), although paced QRS duration is slightly longer (118.09 ± 23.20 vs. 104.26 ± 19.00 ms, *P* = 0.032). It should be noted that the Stim-LVAT of LBBP and LVSP in this study were 48.70 ± 13.67 and 58.70 ± 13.67 ms, respectively, which are significantly shorter than those in any other studies (16). While only one study found no significant difference in Stim-LVAT between LBBP and LVSP (73 ± 15 vs. 81 ± 13 ms, *P* = 0.138), this study proved that LBBP seems to result in a small, but significant, improvement in ventricular synchrony when compared to LVSP by calculating QRS area using electrocardiography and vectorcardiogram (5).

In terms of comparing electrical synchrony between the left and right ventricles, utilizing ultra-high frequency electrocardiography, Curila et al. concluded that LBBP had more interventricular electrical desynchrony than LVSP, although LBBP preserves physiological LV depolarization (12, 13). Jastrzebski et al., in addition to showing that LVSP has longer Stim-LVAT and paced QRS duration than LBBP, established that LVSP has superior interventricular electrical synchrony by comparing the difference of R-wave peak time in V1 and V6 (V6-V1) of LBBP and LVSP (9). Interventricular synchrony is during LVSP improved compared to LBBP, however, at the cost of worsened LV activation. Chen et al. recently employed coronary sinus (CS) electrogram mapping to investigate the difference in LV electrical excitation sequence between LBBP and LVSP (4). In the absence of LBBB, the physiological electrical excitation on the CS electrogram propagates from LV lateral to posterior wall, implying that the ventricular electrogram signal recorded in the distal CS was ahead of the proximal CS. By using CS electrogram mapping, Chen et al. studied 27 LBBP patients and 16 LVSP patients and found that the LV electrical activation sequence of all LBBP were identical to intrinsic rhythm, whereas, that of all LVSP were non-physiological with the earliest activation region changed from lateral to posterior wall (4). Qian et al. first used SPECT imaging to assess ventricular mechanical synchrony in 68 bradycardia patients undergoing LBBP, demonstrating that a constant Stim-LVAT of <76 ms and recorded LBB potential had favorable LV mechanical synchrony and could be used as a criterion to determine LBB capture (7).

## How to differentiating LBBP and LVSP

### Stim-LVAT

Confirming LBB capture is essential for distinguishing LBBP from LVSP. Stim-LVAT, measured at high output pacing and threshold pacing, remained shortest and constant with 100% specificity for determining LBB capture (1, 3). During the decreasing of the pacing output, the transition from NSLBBP to SLBBP or LVSP is frequently observed. The Stim-LVAT remains the shortest and consistent during the transition from NSLBBP to SLBBP if the lead tip is placed in the LBB trunk or its branches, and an isoelectric interval exists between the pacing artifact and the ventricular electrogram signal. If the lead tip is close to the LBB, NSLBBP will be converted to LVSP and Stim-LVAT will be rapidly extended by 10 ms during the output decrease (3). This is because the lead tip may capture both LV septal myocardium and LBB at a high-output pacing voltage, while only LV septal myocardium can be captured at a low-output pacing voltage, resulting in delayed LV electrical activation. However, Shimeno et al. reported that only 41.2% of LBB area pacing showed the output-dependent QRS transition from NSLBBP to SLBBP or LVSP (11).

### LBB potential and retrograde His potential

LBB potential should be recorded in all patients with normal left conduction system (1, 3), but the presence of LBB potential is not direct evidence of LBB capture. In reality, ~68–98% of non-LBBB patients who completed LBBP (6, 39, 42) and a small fraction of those who completed LVSP can record the LBB potential. Chen et al., for example, recorded LBB potential in 88.9% of LBBP and also in 12.5% of LVSP. They assumed that the LBB potential recorded by LVSP was the far-field potentials downstream of LBB, and at this time, a higher pacing output was needed to conduct retrogradely to present a His potential (4). As a result, whether or not LBB potential was recorded could not be utilized as a criterion for LBB capture. The appearance of LBB potential could only indicate that the lead tip was close to the LBB area (3).

Theoretically, for non-LBBB patients, the interval between His potential and LBB potential recorded in sinus rhythm should be same as the interval between LBBP pacing artifact and retrograde His potential (3). At the output was <1.0 V/0.5 ms, retrograde HB and/or anterograde LBB potential recorded from HBP or multielectrode linear catheter which was placed across the aortic valve were evidence of direct LBB capture (3). However, not all patients who complete LBBP can record LBB potential.

## The value of criteria for differentiating LBBP and LVSP

The predictive value of criteria for distinguishing LBBP from LVSP is summarized in Supplementary Table 1. In the non-LBBB group, Wu et al. calculated that Stim-LVAT had a specificity of 95% and sensitivity of 82% for LBB capture at 75 ms, and a specificity and sensitivity of 93 and 76% for LBB capture at 85 ms for LBBB group (3). Jastrzebski et al. reported that the optimal Stim-LVAT value for differentiating between LBBP and LVSP in patients with normal left conduction system was 83 ms, while in patients with LBBB the optimal Stim-LVAT value was 101 ms (6). In addition, they proposed a method to effectively predict LBB capture by combining LBB potential and LVAT in lead V6. For non-LBBB patients, the criterion of “paced LVAT in lead V6 (measured from QRS onset) ≤ native LVAT in lead V6 (+10 ms)” for LBB capture has 98 and 85.7% sensitivity and specificity, respectively. For non-LBBB patients whose LBB potential can be recorded, the sensitivity and specificity of the criterion of “paced Stim-LVAT (measured from stimulus) ≤ LBB potential to LVAT in lead V6 (+10 ms)” for LBB capture were 88.2 and 95.4%, respectively. They also proposed the use of the V6-V1 interval as a discriminating criterion (9). The transition from NSLBBP to SLBBP prolonged the interval of stimulus artifact to late R-wave in lead V1 (RWPTV1; 120.7 ± 16.7 vs. 138.5 ± 21.5 ms, *P* < 0.001), but had no significant effect on Stim-LVAT (77.2 ± 13.6 ms vs. 76.6 ± 14.1 ms, *P* = 0.36). The transition from NSLBBP to LVSP resulted in an increase in Stim-LVAT by ≥15 ms (78.4 ± 10.8 vs. 98.4 ± 13.9 ms), but only an increase of 6.2 ± 6.3 ms in RWPTV1 (119.3 ± 14.5 ms vs. 125.6 ± 13.8 ms, *P* < 0.001). Consequently, during SLBBP, the V6-V1 interval was longest, intermediate during NSLBBP, and shortest during LVSP (62.3 ± 21.4 vs. 41.3 ± 14.0 vs. 26.5 ± 8.6 ms, respectively). The optimal value of V6-V1 interval for distinguishing NSLBBP from LVSP was 33 ms, with a specificity of 90% and a sensitivity of 71.8%, while the V6-V1 interval for confirming the LBB capture was 44 ms, with a specificity of 100% (9). Recently, Chen et al. reported that when LBB potential was recorded in conjunction with Stim-LVAT ≤ 85 ms, the specificity and sensitivity of LBB capture were 93.7 and 95.2%, respectively, whereas if no LBB potential was recorded, Stim-LVAT ≤ 70 ms could also be considered as LBB capture, and vice versa, LVSP (4).

**Figure 1:**
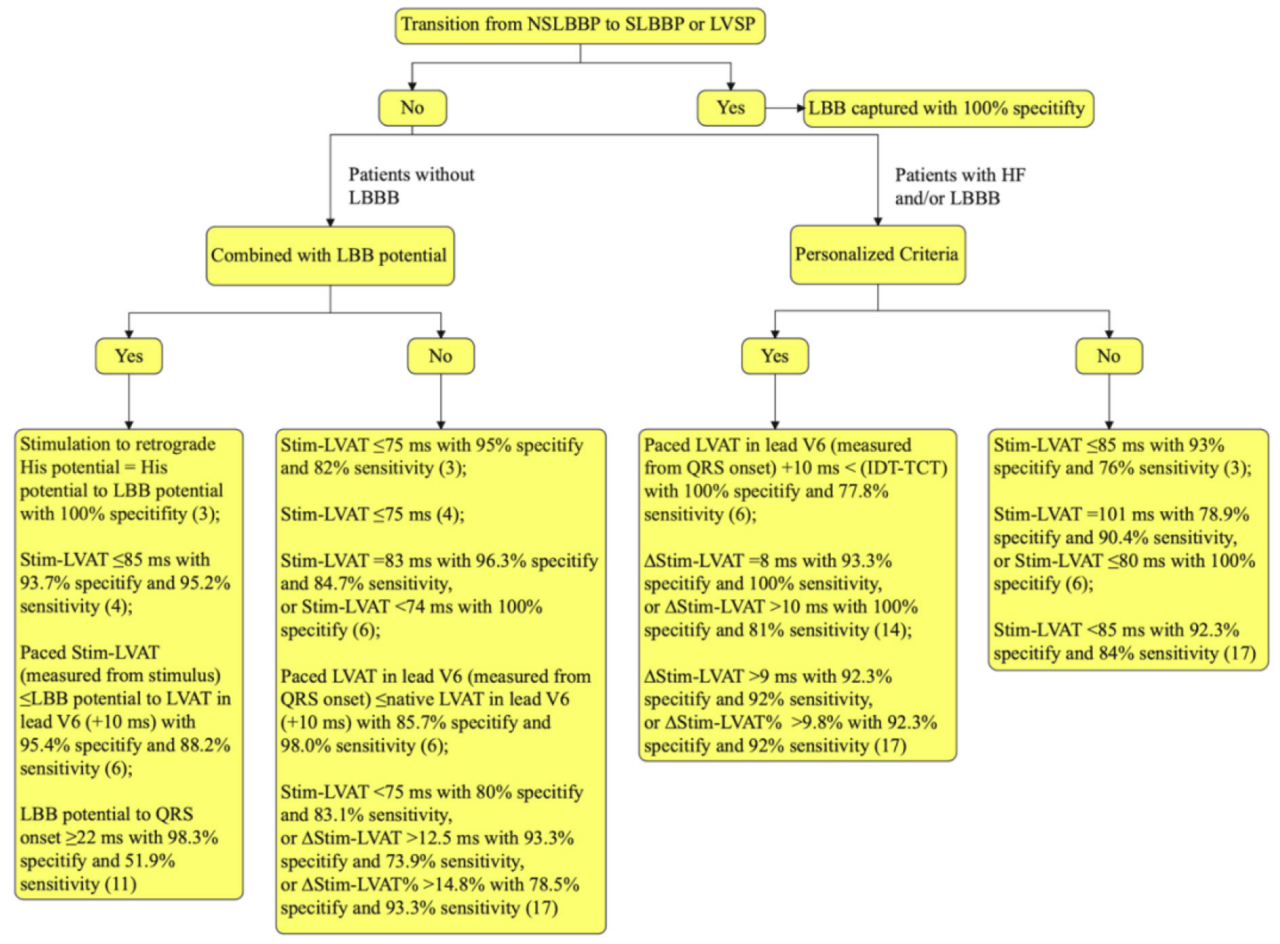
Flowchart for confirming LBB capture. LBB, left bundle branch; LBBB, LBB block; NSLBBP, non-selective LBB pacing; SLBBP, selective LBB pacing; LVSP, left ventricular septal pacing; HF, heart failure; Stim-LVAT, stimulus artifact to left ventricular activation time in lead V5 or V6; LVAT, left ventricular activation time; IDT, the native V6 intrinsicoid deflection time, measured from the earliest QRS onset in any surface lead (global method), not to the point of the highest amplitude, but to the end of the slur/plateau in QRS, that is to the beginning of the final rapid downsloping phase of R wave in lead V6; TCT, the transseptal conduction time, measured from earliest QRS onset in any surface lead to the endocardial indication of the arrival of the depolarization wavefront to the LBB area; ΔStim-LVAT, the Stim-LVAT discrepancy between His bundle pacing and LBBP or LVSP; ΔStim-LVAT%, ΔStim-LVAT divided by Stim-LVAT of His bundle pacing.

Stim-LVAT varies across patient populations and is prolonged in large ventricular size or LBBB in patients with heart failure (HF) (17), suggesting that determining LBB capture with a fixed value of Stim-LVAT is challenging. As a result, a personalized criterion to confirm LBB capture is beneficial. Jastrzebski et al. used the difference between the native V6 intrinsicoid deflection time (IDT) and the transseptal deflection time (TCT) to predict the LBB capture. The sensitivity and specificity of “paced LVAT in lead V6 (measured from QRS onset) +10 ms <(IDT-TCT)” for confirming LBB capture in LBBB patients were 77.8 and 100%, respectively (6). Vijayaraman et al. determined the Stim-LVAT of LBBB patients during HBP, NSLBBP, SLBBP, and LVSP and found it to be 91.7 ± 8.4, 75.2 ± 8.8, 76.9 ± 8.6, and 90.4 ± 9.1 ms, respectively (*P* < 0.001; LBBP vs. HBP and LVSP) (14). The delta Stim-LVAT between HBP and LBBP or LVSP (ΔStim-LVAT) was then used to confirmed the LBB capture. When ΔStim-LVAT was 8 ms, the specificity and sensitivity of LBB capture in LBBB patients were 93.3 and 100%, respectively. When >10 ms, 100 and 81% (14). Recently, Qian et al. established that ΔStim-LVAT may be utilized as a reliable criterion to distinguish LBBP from LVSP in HF patients with or without LBBB. A cut-off value of ΔStim-LVAT > 9 ms confirmed LBB capture with 92% sensitivity. Furthermore, the percent reduction in ΔStim-LVAT, ΔStim-LVAT divided by Stim-LVAT of HBP (ΔStim-LVAT%), also shows excellent accuracy for LBB capture (17). The flowchart for confirming LBB capture in patients is summarized in Figure 1.

## Conclusion

The transition from NSLBBP to SLBBP or LVSP is the gold standard for confirming LBB capture, but it may not be achieved due to similar capture thresholds for LBB and nearby myocardium. In this scenario, the recorded LBB potential combined with short Stim-LVAT can predict LBB capture and ventricular mechanical synchrony better. Personalized criteria, such as ΔStim-LVAT, ΔStim-LVAT%, or comparison of paced LVAT and difference between IDT and TCT, can be utilized to confirm LBB capture in patients with HF or LBBB.

## Author contributions

KZ: conceptualization and writing—original draft preparation. LL and JL: contribute to our revised draft and provide useful comments. DC and QL: supervision and writing—reviewing and editing. All authors contributed to the article and approved the submitted version.

## Funding

This work was supported by research grant No. 3502Z20214ZD1165 from Xiamen Municipal Bureau of Science and Technology.

## Conflict of interest

The authors declare that the research was conducted in the absence of any commercial or financial relationships that could be construed as a potential conflict of interest.

## Publisher's note

All claims expressed in this article are solely those of the authors and do not necessarily represent those of their affiliated organizations, or those of the publisher, the editors and the reviewers. Any product that may be evaluated in this article, or claim that may be made by its manufacturer, is not guaranteed or endorsed by the publisher.
