# Corrigendum: Criteria for differentiating left bundle branch pacing and left ventricular septal pacing: a systematic review

**DOI:** 10.3389/fcvm.2024.1404850

**Published:** 2024-04-26

**Authors:** Kailun Zhu, Linlin Li, Jianghai Liu, Dong Chang, Qiang Li

**Affiliations:** ^1^ Department of Cardiology, Xiamen Cardiovascular Hospital of Xiamen University, School of Medicine, Xiamen University, Xiamen, China; ^2^ School of Medicine, Xiamen University, Xiamen, China

**Keywords:** left bundle branch pacing, left ventricular septal pacing, QRS complex, electrocardiogram, electrophysiology

A Corrigendum on Criteria for differentiating left bundle branch pacing and left ventricular septal pacing: a systematic review By Zhu K, Li L, Liu J, Chang D and Li Q (2022). Front. Cardiovasc. Med. 9:1006966. doi: 10.3389/fcvm.2022.1006966

In the published article, there was an error. In the transition from NSLBBP to SLBBP and the transition from NSLBBP to LVSP, the description of RWPTV1 and Stim-LVAT was incorrect.

A correction has been made to **The value of criteria for differentiating LBBP and LVSP**, paragraph 1. This sentence previously stated:

“The transition from NSLBBP to SLBBP prolonged the interval of stimulus artifact to late R-wave in lead V1 (RWPTV1; 120.7 ± 16.7 vs. 138.5 ± 21.5 ms, *P *< 0.001) and Stim-LVAT (119.3 ± 14.5 vs. 125.6 ± 13.8 ms, *P *< 0.001). The transition from NSLBBP to LVSP resulted in an increase in Stim-LVAT by ≥15 ms (78.4 ± 10.8 vs. 98.4 ± 13.9 ms) but only minimally influenced RWPTV1 (77.2 ± 13.6 vs. 76.6 ± 14.1 ms, *P* = 0.36)”.

The corrected sentence appears below:

“The transition from NSLBBP to SLBBP prolonged the interval of stimulus artifact to late R-wave in lead V1 (RWPTV1; 120.7 ± 16.7 vs. 138.5 ± 21.5 ms, *P *< 0.001), but had no significant effect on Stim-LVAT (77.2 ± 13.6 ms vs. 76.6 ± 14.1 ms, *P* = 0.36). The transition from NSLBBP to LVSP resulted in an increase in Stim-LVAT by ≥15 ms (78.4 ± 10.8 vs. 98.4 ± 13.9 ms), but only an increase of 6.2 ± 6.3 ms in RWPTV1 (119.3 ± 14.5 ms vs. 125.6 ± 13.8 ms, *P* < 0.001)”.

The authors apologize for this error and state that this does not change the scientific conclusions of the article in any way. The original article has been updated.

