# Supplementary material for: Criteria for differentiating left bundle branch pacing and left ventricular septal pacing: A systematic review
Source: Front Cardiovasc Med. 2022 Sep 30;9:1006966. doi: 10.3389/fcvm.2022.1006966 (PMC9562849; doi:10.3389/fcvm.2022.1006966)
Supplement: Supplementary file 1 [file Table_1.docx]

***Supplementary Table* Criteria for differentiating LBBP and LVSP**

| Study (Year) | Patient populations | Criteria for differentiation between LBBP and LVSP | Predictive value | | |
| --- | --- | --- | --- | --- | --- |
|  |  |  | Specificity | Sensitivity | |
| Qian et al.  (2020) (7) | All patients | Stim-LVAT <76 ms predict the normal LV mechanical synchrony | 87.5% | 88.9% | |
|  |  | Stim-LVAT <76 ms with LBB potential predict the  normal LV mechanical synchrony | 100% | 77.8% | |
| Jastrzębski et al. (2020) (6) | Non-LBBB | Stim-LVAT =83 ms | 96.3% | 84.7% | |
|  |  | Stim-LVAT <74 ms | 100% | none | |
|  |  | Paced LVAT (measured from QRS onset) ≤native LVAT in lead V6 (+10 ms) | 85.7% | 98% | |
|  |  | Paced Stim-LVAT (measured from stimulus) ≤LBB potential to LVAT (+10 ms) | 95.4% | 88.2% | |
|  | LBBB | Stim-LVAT =101 ms | 78.9% | 90.4% | |
|  |  | Stim-LVAT ≤80 ms | 100% | none | |
|  |  | Paced LVAT in lead V6 (measured from QRS onset) +10ms <(IDT-TCT) | 100% | 77.8% | |
| Wu et al.  (2021) (3) | Non-LBBB | Stim-LVAT ≤75 ms | 95% | 82% | |
|  | LBBB | Stim-LVAT ≤85 ms | 93% | 76% | |
| Vijayaraman et al. (2021) (14) | LBBB | ∆Stim-LVAT =8 ms | 93.3% | 100% | |
|  |  | ∆Stim-LVAT >10 ms | 100% | 81% | |
| Jastrzębski et al. (2021) (9) | All patients | V6-V1 >33 ms | 90% | 71.8% | |
|  |  | V6-V1 >44 ms | 100% | none | |
| Chen et al  (2022) (4) | Patients without LBBB | Stim-LVAT ≤85 ms  with LBB potential | 93.7% | 95.2% | |
|  |  | Stim-LVAT ≤70 ms  with No LBB potential | None | | None |
| Shimeno et al. (2022) (11) | All patients | LBB potential to QRS onset ≥22 ms | 98.3% | 51.9% | |
|  |  | Stim-LVAT <68 ms | 67.8% | 78.8% | |
| Qian et al.  (2022) (17) | Non-HF | Stim-LVAT <75 ms | 80.0% | 83.1% | |
|  |  | ∆Stim-LVAT >12.5 ms | 93.3% | 73.9% | |
|  |  | ∆Stim-LVAT% >14.8% | 78.5% | 93.3% | |
|  | HF | Stim-LVAT <85 ms | 92.3% | 84% | |
|  |  | ∆Stim-LVAT >9 ms | 92.3% | 92% | |
|  |  | ∆Stim-LVAT% >9.8% | 92.3% | 92% | |

LBBP, left bundle branch pacing; LVSP, left ventricular septal pacing; LVAT, left ventricular activation time; IDT, the native V6 intrinsicoid deflection time, measured from the earliest QRS onset in any surface lead (global method), not to the point of the highest amplitude, but to the end of the slur/plateau in QRS, that is to the beginning of the final rapid downsloping phase of R wave in lead V6; TCT, the transseptal conduction time, measured from earliest QRS onset in any surface lead to the endocardial indication of the arrival of the depolarization wavefront to the LBB area; Stim-LVAT, stimulus artifact to left ventricular activation time in lead V5 or V6; LV, left ventricular; LBB, left bundle branch; LBBB, left bundle branch block; ∆Stim-LVAT, the Stim-LVAT discrepancy between HBP and LBBP or LVSP; V6-V1, difference of R-wave peak time in V1 and V6; HF, heart failure; ∆Stim-LVAT%, ∆Stim-LVAT divided by Stim-LVAT of His bundle pacing.
